# Expression and Clinical Significance of Thyroid-stimulating hormone receptor in the Subtypes of Papillary thyroid carcinomas

**DOI:** 10.12669/pjms.39.1.6096

**Published:** 2023

**Authors:** Wei Shen, Shanshan Zhang, Qinghuai Li

**Affiliations:** 1 Wei Shen, Department of Thyroid and Breast Surgery, The Second Hospital of Hebei Medical University, 215 Peace West Road, Shijiazhuang 050000, Hebei, P.R. China; 2 Shanshan Zhang, Department of Thyroid and Breast Surgery, The Second Hospital of Hebei Medical University, 215 Peace West Road, Shijiazhuang 050000, Hebei, P.R. China; 3 Qinghuai Li, Department of Thyroid and Breast Surgery, The Second Hospital of Hebei Medical University, 215 Peace West Road, Shijiazhuang 050000, Hebei, P.R. China

**Keywords:** Subtype of papillary thyroid carcinoma, Thyroid-stimulating hormone receptor, Immunohistochemical staining, TSH suppression therapy

## Abstract

**Objective:**

To investigate the expression of TSH receptors (TSHR) in various subtypes of Papillary thyroid carcinomas (PTC) by immunohistochemistry.

**Methods:**

Retrospective analyses were carried out to the clinical data of 108 PTC patients randomly admitted into the Department of Thyroidthyroid surgery thyroid surgery and Breast Surgery, The Second Hospital of Hebei Medical University from March 2020 to December 2020. The archived paraffin blocks of the 108 cases as well as 18 contiguous normal thyroid tissues (control group) were taken from the Department of Pathology of The Second Hospital of Hebei Medical University. The pathological types of all PTC tissues were detected and the expression of TSHR was determined.

**Results:**

TSHR expression was 86.11% positive in PTC tissues; with 85.00% positive in classical group; with 75.86% positive in micro group; with 84.61% positive in follicular group; with 83.33% positive in oncocytic group; with 50.00% positive in invasive group. TSHR expression was 100% in normal thyroid tissues. So TSHR expression in normal thyroid tissues is significantly higher than that in PTC; TSHR expression in microcarcinoma is stronger than in the other subtypes; there is no significant difference among the other subtypes.

**Conclusions:**

TSH suppression works better on microcarcinoma than on the other subtypes. And the effects on non-invasive subtypes are better than on invasive subtypes.

## INTRODUCTION

Papillary thyroid carcinoma (PTC) accounts for more than 90% of all thyroid cancer cases. The heteromorphism of PTC cells is small, so the surgical treatment effect is good and the patient prognosis is good. Studies suggest that those cases with strong invasiveness and poor prognosis mostly belong to individual subtypes of PTC.^1^,^2^ The subtype is an important factor of determining the biological behavior of PTC and affecting the prognosis of PTC patients. It is of far-reaching significance to distinguish and study the pathophysiological mechanisms and clinical manifestations of different PTC variants.

For postoperative PTC patients, superphysiological doses of thyroid hormone (TH) shall be applied to inhibit thyroid stimulating hormone (TSH) to a normal lower limit or below the lower limit or even to an undetectable level.^3^ This makes TSH suppression an important therapeutic measure after PTC operation. However, whether the inhibitory effect of TSH control on PTC is effective remains controversial in academia. In order to reduce the occurrence of the side effects of TSH suppression, it is important to apply individualized TSH suppression to PTC patients.^4^ TSH takes effect after binding with its receptor (TSHR). Studies have shown that the expression of TSHR is positively correlated with the differentiation degree of thyroid cancer.^5^,^6^

Therefore, it is of great significance to undertake appropriate treatment according to the histologic appearance of PTC, TSHR expression, and response to treatment. This experiment investigates the expression of TSHR in various pathological subtypes of PTC, and explores the possible relationship between the efficacy of endocrinotherapy and the pathological subtypes of PTC to provide a theoretical basis for the clinical formulation of individualized TSH suppression protocols for PTC patients.

## METHODS

Retrospective analyses were carried out to the clinical data of 108 PTC patients randomly admitted into the Department of Thyroidthyroid surgery thyroid surgery and Breast Surgery, The Second Hospital of Hebei Medical University from March 2020 to December 2020. The archived paraffin blocks of the 108 cases as well as 18 contiguous normal thyroid tissues (control group) were taken from the Department of Pathology of The Second Hospital of Hebei Medical University. None of the patients received any preoperative treatment associated with thyroid diseases. All the tissue specimens had complete clinical medical records and pathological data. The study was approved by the Institutional Ethics Committee of The Second Hospital of Hebei Medical University on January 27, 2021 (No. [2021] R046), and written informed consent was obtained from all participants.

All thyroid tissue sections were confirmed by two pathologists. WHO standard^7^: PTC with a diameter of ≤1.0 cm is called “thyroid papillary microcarcinoma”; PTC with a typical PTC cell nucleus, over 50% of follicular structure and no well differentiated papillary structure is called “follicular subtype of PTC”; oncocytic subtype is a papillary carcinoma composed of oncocytic cells, whose nucleus has the characteristics of papillary carcinoma? Pathological characteristics of insular carcinoma: tumor cell nests or “insula” compose a unique appearance, with little cytoplasm; regional lymph nodes and distant metastases are common. Pathological characteristics of Tumor cells may diffusely infiltrate unilateral or bilateral thyroid glands; lymphatic vessels and blood vessels are commonly infiltrated, with a large amount of lymphocyte infiltration; fibrosis is obvious; small papillary structures and a large number of psammoma bodies can be seen in lesions; and squamous intestinal metaplasia can be seen around the papillary structures. The latter two are collectively known as “invasive subtypes”. After non-specific staining and background staining were excluded from the immunohistochemical staining results, the pale yellow to brown of the cell membrane and cytoplasm of the thyroid tissue suggested “positive”.

The sum of the cell staining intensity score and the cell positive percentage score was determined semi quantitatively. Two pathologists randomly selected five high power fields (10×40) for observation. Pale yellow: one point, brownish yellow: two points, yellowish brown: three points, and no staining: zero point; when 100 cells were counted under each high-power field, positive rate <five%: zero-point, 5%~25%: one point, 26%~50%: two points, above 50%: 3 points. After the two scores were added up, “0 point” means negative (-), “one-two points” means weakly positive (+), “three-four points” means positive (++), and “five-six points” means strongly positive (+++). Data analysis was performed with chi-square test, non-parametric test and rank correlation in SPSS21.0.

## RESULTS

**Fig.1:**
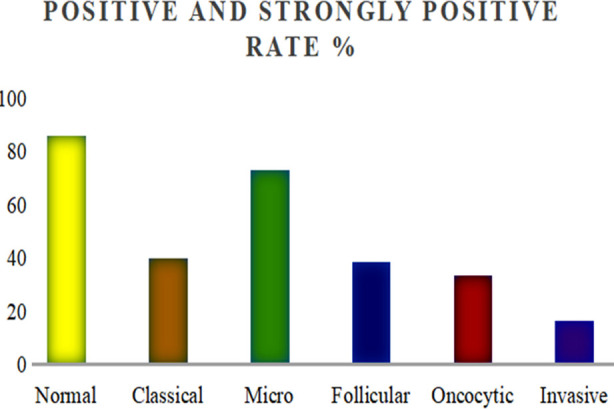
Thyroid papillary carcinoma subtype and normal thyroid tissue TSHR positive and strong positive expression rate in comparison.

According to observation and analysis of HE routine staining, among the 108 PTC sections studied in this experiment, 40 belonged to classical subtype, accounting for 37.03%; 37 belonged to microcarcinoma, accounting for 34.26%; 13 belonged to follicular subtype, accounting for 12.04%; 12 belonged to oncocytic subtype, accounting for 11.11%; 5 belonged to insular subtype, accounting for 4.63%; and one belonged to diffuse sclerosis subtype, accounting for 0.92%. Insular and diffuse sclerosis subtypes are collectively known as “invasive subtypes”.Fig-1.

Data analysis was done of 108 cases with complete clinical records. A. Gender: 26 males, 82 females, male: female≈1:3; B. Age: onset age: 13 to 80; C. Treatment: lymphadenectomy was performed by the intraoperative frozen section pathological results of “PTC”; D. Number of central lymph node metastasis cases: 61 cases, percentage: ≈56.48%. The microcarcinoma central lymph node metastasis cases accounted for about 28.85%, taking up 47.57% of the total number of microcarcinoma cases; the central lymph node metastasis ratio of invasive subtype was 100%.

**Fig.2:**
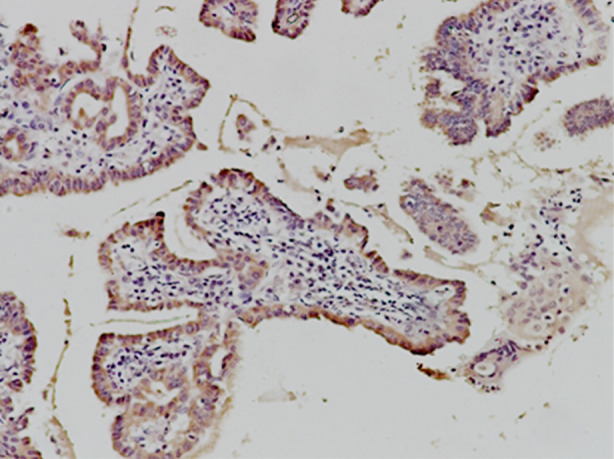
Classic thyroid papillary carcinoma TSHR positive staining 400X.

Expression of TSHR in classical subtype, microcarcinoma, follicular subtype, oncocytic subtype, insular subtype, diffuse sclerosis subtype and contiguous normal thyroid tissue. TSHR is mainly expressed in basement membrane in normal thyroid tissue. It was expressed in the cytomembrane and cytoplasm of all subtypes of PTC studied in this experiment, as shown in Fig-2. Non-invasive subtypes include classical, micro, follicular, and oncocytic subtypes; invasive subtypes include insular and diffuse sclerosis subtypes.

Among the 108 PTC tissue specimens, the positive rate was 86.11%, and the positive and strongly positive rate accounted for 49.07%. The five subtypes of PTC were different in TSHR expression, as shown in Table-I. TSHR expression in contiguous normal thyroid tissue was higher than that in any subtype and that TSHR expression in microcarcinoma was higher than that in any other subtype. There were no significant differences among the other subtypes.

**Table-I:** The expression of TSHR in classical and microcarcinoma, follicular, oncocytic, PTC with focal insular component variant of papillary thyroid carcinoma.

Pathological type	—	+	++	+++	n	Positive rate	R
Classical	6	18	14	2	40	85.00%	50.75α
Micro	2	8	16	11	37	94.59%	75.86β
Follicular	2	6	3	2	13	84.61%	53.58γ
Oncocytic	2	6	4	0	12	83.33%	45.75δ
Invasive	3	2	1	0	6	50.00%	29.25ε
Normal tissue	0	2	4	12	18	100%	96.83θ

Total	15	42	42	27	126	—	—

χ^2^=36.295, df=5, P<0.001.

The TSHR positive rate of non-invasive subtypes was 88.24%, while the TSHR positive rate of invasive subtypes was 50%. There was a statistically significant difference between the two groups, with higher TSHR expression in non-invasive subtypes, as shown in Table-II.

**Table-II:** The relationship between the expression of TSHR and degree of invasiveness of papillary thyroid carcinoma.

Degree of invasiveness	—	+	++	+++	N	Positive rate	R
Non-invasive	12	38	37	15	102	88.24%	56.04
Invasive	3	2	1	0	6	50%	28.25

Total	15	40	38	15	108	—	—

Z= -2.226,*P*=0.026.

## DISCUSSION

Classical PTC features complex papillary structure and typical cell nucleus morphology, that is, frosted glass nucleus, nuclear grooves, and pseudo-inclusion bodies. Compared with the classical subtype, a few subtypes show stronger invasiveness, high rates of extragladular infiltration, common distant metastases, and insensitivity to radioactive iodine, severely threatening the prognosis of patients. Besides classical subtype, PTC is classified into 15 subtypes in WHO classification.^7^,^8^ This study only involves six common subtypes clinically. Surgical resection is a main treatment means for differentiated thyroid carcinoma. However, not all the patients benefit from surgeries. Studies show that prophylactic lymphadenectomy cannot improve the long-term prognosis of some patients and instead increases the risk of postoperative hypocalcemia and bilateral recurrent laryngeal nerve injury.^9^-^11^Therefore, the performance of prophylactic central lymphadenectomy depends on the professional technical level of the operator. Skilled specialists can even reduce the incidence of thyroid surgery complications to below 1%.^12^,^13^

The principle of TSH suppression therapy is drawing on hormone increasing plasma T3 and T4 concentrations to normal upper limit, inhibit the synthesis and release of TSH, so as to inhibit tumor growth and metastasis.^14^,^15^ It has been reported that continuous high levels of thyroid hormone may significantly increase the occurrence of cardiovascular and other adverse events in patients aged >60.^16^,^17^ In addition, literature has reported that TH-based suppression therapy brings a higher risk of adverse reactions to specific groups, including AF, especially in patients with concurrent left ventricular volume dilation, cerebral infarction or transient ischemic attacks, heart failure, heart valvular diseases, osteoporosis or previous fractures, diabetes, and renal failure.^18^,^19^ The efficacy evaluation of suppression therapy, whether all PTC patients need to receive suppression therapy and to what extent remain controversial. Therefore, the differentiated side effects and efficacy of suppression therapy make it imperative to administer individualized endocrinotherapy to PTC patients.

Studies suggest that detecting TSHR expression can help formulate a suppression regimen and predict the efficacy of suppression therapy.^20^,^21^ Theoretically speaking, the effect of suppression therapy is good for tumors with high TSHR expression, while the effect is poor or nil for tumors with low expression or almost no expression, where an alternative of thyroid hormone is needed. Comprehensive genetic and pathomorphological diagnosis can help us distinguish between patients with high inhibitory treatment benefits and patients with low inhibitory treatment benefits.^22^ Then, is there differential TSHR expression among the subtypes of PTC? If so, individualized treatment based on the recurrence and receptor expression of each subtype can effectively prevent tumor growth and recurrence, and reduce the side effects of TSH suppression therapy, thus prolonging the survival time and improving the quality of life for patients after operation and maximizing the interests of patients. Related studies indicate that the efficacy of suppression therapy is positively correlated with TSHR expression. This study infers that suppression therapy works better on the non-invasive group, particularly microcarcinoma, than on the other subtypes. Then, detecting TSHR expression in PTC patients can be used as an indirect predictor of suppression therapy efficacy. In this experiment, the distribution and expression of TSHR in different subtypes of PTC were studied by immunohistochemistry, and we found that the expression of TSHR was higher in non-invasive subtypes than in invasive subtypes. Especially, the expression in microcarcinoma was significantly higher than that in the other subtypes. Pathological subtypes are another factor affecting patients’ prognosis. In particular, invasive subtypes feature strong invasiveness and high incidences of metastasis and recurrence. According to the statistics of this experiment, invasive subtypes had a central lymph node metastasis rate of 100%; central lymph node metastasis was also common in microcarcinoma, suggesting the universal applicability of prophylactic central lymph node dissection in PTC patients. Considering TSHR plays an important role in the occurrence and development of thyroid cancers, the in-depth study of TSHR is of great significance to exploring the diagnosis and treatment of thyroid cancers. Our results provide data support for the clinical efficacy of TSHR endocrine therapy for different Subtypes of PTC.

### Limitations of this study:

This experiment is based on theoretical research, without clinical observation on the relationship between TSHR expression and suppression effect. Besides, the sample size is small. So clinical trials of large sample sizes are needed for further verification.

## CONCLUSION

TSHR expression was 100% in normal thyroid tissue, significantly stronger than in PTC. The endocrine therapy works better on microcarcinoma than on the other subtypes, and the efficacy on the other subtypes is similar. Suppression therapy works better on non-invasive PTC subtypes than on invasive subtypes.

### Authors’ Contributions:

**WS** designed this study, prepared this manuscript are responsible and accountable for the accuracy and r integrity of the work.

**QL** collected and analyzed clinical data.

**SZ** Data analysis and significantly revised this manuscript.
